# Transposable Elements in Human Cancer: Causes and Consequences of Deregulation

**DOI:** 10.3390/ijms18050974

**Published:** 2017-05-04

**Authors:** Sumadi Lukman Anwar, Wahyu Wulaningsih, Ulrich Lehmann

**Affiliations:** 1 Division of Surgical Oncology, Department of Surgery Faculty of Medicine, Universitas Gadjah Mada, Yogyakarta 55281, Indonesia; 2 Institute of Pathology, Medizinische Hochschule Hannover, Hannover 30625, Germany; 3 PILAR (Philippine and Indonesian Scholar) Research and Education, 20 Station Road, Cambridge CB1 2JD, UK; w.wulaningsih@ucl.ac.uk; 4 MRC (Medical Research Council) Unit for Lifelong Health and Ageing, University College London, London WC1B 5JU, UK; 5 Division of Haematology/Oncology, Faculty of Medicine Universitas Gadjah Mada, Yogyakarta 55281, Indonesia

**Keywords:** transposable elements, epigenetics, cancer, genomic instability, non-coding RNAs

## Abstract

Transposable elements (TEs) comprise nearly half of the human genome and play an essential role in the maintenance of genomic stability, chromosomal architecture, and transcriptional regulation. TEs are repetitive sequences consisting of RNA transposons, DNA transposons, and endogenous retroviruses that can invade the human genome with a substantial contribution in human evolution and genomic diversity. TEs are therefore firmly regulated from early embryonic development and during the entire course of human life by epigenetic mechanisms, in particular DNA methylation and histone modifications. The deregulation of TEs has been reported in some developmental diseases, as well as for different types of human cancers. To date, the role of TEs, the mechanisms underlying TE reactivation, and the interplay with DNA methylation in human cancers remain largely unexplained. We reviewed the loss of epigenetic regulation and subsequent genomic instability, chromosomal aberrations, transcriptional deregulation, oncogenic activation, and aberrations of non-coding RNAs as the potential mechanisms underlying TE deregulation in human cancers.

## 1. Introduction

Over two-thirds of the human genome is composed of repetitive sequences [1]. Transposable elements (TEs), which make up the majority of repetitive sequences (up to 50% of the human genome), can jump within the genome and play an important role as an engine for the dynamics of human evolution and the pathogenesis of human cancers [2]. TEs are classified according to their transposition mechanisms into retrotransposons (Class I) and DNA transposons (Class II) [3,4]. Class I retrotransposons are further sub-classified into long terminal repeats (LTRs) or endogenous retrovirus (ERV) elements that make up 8% of the human genome, and non-LTRs, which include the two most important TEs in cancers, long interspersed nuclear elements (LINEs) and short interspersed nuclear elements (SINEs) [3,4]. LTRs are identical repeated DNA sequences originating from the integration of ancient retroviruses into the human genome that have lost their ability as mobile elements. On the other hand, non-LTRs with autonomous and nonautonomous retrotranspositions (LINEs and SINEs, respectively) retain their ability as active mobile elements in the human genome. *LINE1* sequences represent up to 17% of the human genome [3,4]. Although *LINE1* is considered the most active mobile element in a human, around 500,000 copies no longer have the ability to mediate retrotransposition due to significant *LINE1* truncations [5]. However, new full length *LINE1* would be able to encode proteins that are very efficient to “copy and paste” into a new genetic location and hamper the associated gene expression and/or drive oncogenic process through the transcription of chimeric proteins [5].

Active TEs are considered highly mutagenic and are associated with the multiple steps of cancer development and progression [2,6]. TEs have been demonstrated to play an active role in regulating the human genome by governing endogenous gene expression, as well as generating novel genetic loci [7]. However, TE activity might have different impacts on the human genome ranging from positive to negative consequences, including the maintenance of centromere and telomere integrity, recombinant genome remodeling, and deleterious gene expression [3,7]. In addition, the accumulation of TEs throughout human evolution has been adapted into novel functions through several mechanisms, known as domestication. The co-opting of TEs can be delivered through several ways such as the formation of a new gene entity, integration into an existing gene generating a chimeric protein, and insertion into a regulatory region upstream of a gene to further regulate gene expression by forming alternative promoters or altering transcriptional binding sites [8,9]. In addition, the integration of TEs into introns might interfere with transcription, alternative-splicing, polyadenylation, and messenger RNA stability [3,10,11].

A growing body of evidence has documented the essential role of TEs in human carcinogenesis. The insertion of TEs into genes that are responsible for DNA repair including *BRCA2* [12], *APC* [13], and *RB1* [14] can cause the disruption of gene expression and further affect genome instability [15]. Methylation loss of a specific *LINE1* promoter is able to activate an alternative transcript that encodes a truncated and constitutively active MET protein in bladder cancer [16]. De novo insertions of LTR and *LINE* sequences cause an alternative transcription of a new isoform in an *ALK* (anaplastic lymphoma kinase) gene [17]. The new *ALK* isoform is specifically expressed in around 11% of melanomas that further show a specific response to the ALK inhibitor [17]. A comprehensive approach in diffuse large B-cell lymphomas (DLBCLs) has detected multiple LTR transcripts in several genes, including fatty-acid binding protein 7 (*FABP7*); that are known to promote lymphomagenesis [18]. The reactivation of ancient LTR has also been associated with the novel oncogenic transcription of *ERBB4* in ALK-negative anaplastic large cell lymphoma (ALCL) [19]. Two aberrant *ERBB4* transcripts are found in almost a quarter of ALK-negative ALCL patients [19]. Despite their important roles in genome regulation, the detailed mechanisms of TE reactivation in tumor development remain largely unexplained.

TEs are tightly regulated from early embryonic development and during the entire human life [20]. Epigenetic mechanisms, particularly DNA methylation and histone modifications, are the best known mechanisms underlying the repression and regulation of TEs [3,21]. In relation to human cancers, epigenetic alterations have also been continuously reported to play a significant role in the initiation of tumor development [22]. A growing number of studies have delineated that epigenetic mechanisms may also control TE reactivation with subsequent effects on carcinogenesis [2,21]. The hypermethylation of tumor suppressor genes accompanied by global hypomethylation occurs consecutively in human cancers [22,23]. Moreover, the global loss of methylation subsequently results in the reactivation of TEs [3,21,24]. In cancers, hypomethylation and TE activation are dynamic processes during tumor evolution and progression [25,26,27]. The reactivation of TEs could initiate oncogene activation [16,25], chromosomal breakages [28], and genomic instability [27] that further contribute to tumor initiation and progression [16,27]. To obtain a better understanding of this emerging area of research, we reviewed the current evidence on the deregulation of TEs by epigenetic mechanisms, especially DNA methylation and non-coding RNAs, and their potential contribution to the development of human cancers through genomic instability, chromosomal aberrations, and oncogenic activation.

## 2. Widespread Epigenetic Deregulation of Repetitive Elements in Cancer

**Figure 1:**
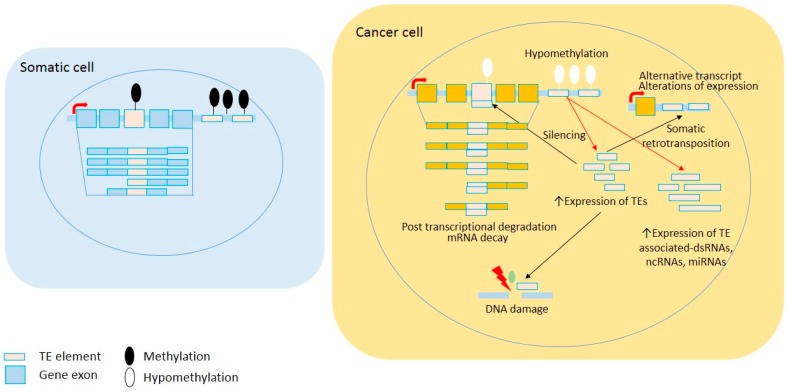
Global loss of DNA methylation in cancer cells leads to TE reactivation. A common epigenetic feature in neoplastic cells is global demethylation, including within repeated sequences. Subsequently, TE reactivation can cause increasing somatic retrotransposition, non-coding RNA, and transcriptional deregulation. Red arrows show direct impacts of TE reactivation and black arrows show effects of retrotransposition.

TEs have been suggested to play an essential role in maintaining genome integrity [29] and therefore, dysregulated TE activity may result in genomic instability and subsequent carcinogenesis [5,30]. TEs and host regulatory factors cooperate in controlling TE activity through overlapping multiple epigenetic mechanisms [31]. TEs are able to independently recruit silencing signals to form robust building blocks of inactivation not only at the level of a single gene, but also across large chromosomal regions [3,31]. In addition, self-control mechanisms by TEs will initiate the repression of retrotransposition adverse effects while maintaining their ability to replicate. The interdependency of host regulatory controls is mediated through the transcription of truncated suppressor clones, the presence of splice sites and hidden polyadenylation signals, and transposon-mediated autoregulation (as reviewed in [32]). Several other host regulatory defense mechanisms are also involved in restricting TE activity. However, the primary regulators controlling TE transcription are epigenetic mechanisms, mainly DNA methylation and histone modifications; that effectively suppress TEs while maintaining their presence in the human genome [3,31]. In the context of cancer, the loss of DNA methylation causing the reactivation of *LINE1* has been reported in colorectal cancer [26], hepatocellular carcinoma [33], and breast cancer [34]. Figure 1 describes the effects of the loss of methylation on TE reactivation in a cancer cell.

The hypomethylation of *LINE1* is reported in both solid tumors and leukemia, and is frequently correlated with unfavorable clinical outcomes [26,35]. In addition, the hypomethylation of *LINE1* and *Alu* in circulating blood has potential value for cancer diagnoses [36]. Exposure to DNA demethylation agents including azacytidine and decitabine leads to *LINE1* demethylation, as well as subsequent activation, and is followed by oncogene activation [16]. The human endogenous retroviruses (HERVs) are also related to carcinogenesis. Increased levels of HERV RNA and reverse transcriptase enzymes have been reported in lymphomas and breast cancers [37]. In addition, HERV-like-viruses have also been associated with melanomas, especially those with metastases [37]. Ovarian, colon, and testicular cancers express higher levels of envelope genes of HERV [37].

Heterochromatin formation has also been associated with the regulation of TE silencing in cooperation with DNA methylation and small RNAs [31]. Histone tail modifications affect the binding of protein and transcription factors. TE-associated nucleosomes are commonly methylated at the histone 3 lysine 9 (H3K9) representing signals for transcriptional silencing and inactive chromatin [38]. Mutations of H3K9 methyltransferases including *SUV39* cause TE upregulation [39,40]. In addition, the deregulation of DNA methyltransferase during embryonic development leads to increased TE activity and is associated with some developmental disorders [31,41]. Specific patterns of DNA sequences and non-coding RNAs are implicated in the direct de novo and maintenance DNA methylation of specific genomic loci, including repetitive sequences [22,31]. Chromatin structure-modifying proteins implicated in chromatin remodeling and condensation are also involved in TE silencing [31,42]. Several TEs, particularly SINEs, contain binding sites for CTCF that function as an insulator and consecutively regulate chromatin condensation [43,44]. A recent study by Pugacheva et al. showed the specific binding of CTCF and BORIS to DNA repeats in which BORIS preferentially bound to SVA repeats (*SINE*s, *VNTR*s, *Alu*s), suggesting a potential role of BORIS in the regulation of active TEs in cancer cells [45]. DDM1, LSH1, SETDB1, and KDM1A are among numerous chromatin remodeling proteins that regulate and maintain TE inactivation [46]. Especially in mammals, DNA methylation, along with chromatic remodeling factors and non-coding RNAs, are responsible for keeping TEs in a dormant state [42]. However, recent evidence suggests that different mechanisms are involved in regulating TEs during embryogenesis, mainly through using histone modifying enzymes such as KDM1A and SETDB1 [21,46]. Upon epigenetic silencing, TEs are kept inactive to allow their embedded regulatory regions to be exapted. Mutations and the deregulation of chromatin remodeling genes are closely related with human cancers of different etiologies [47,48]. However, their direct effects on the reactivation of repetitive elements in cancers to initiate genomic instability and oncogenic activation need to be clarified [49,50].

## 3. Transcriptional Deregulation

TEs are able to modulate gene transcription by insertion into transcriptional regulatory regions [3]. TE insertions can induce functional defects of regulatory regions, including promoters, enhancers, or silencers [3]. TE integrations are able to create new exons that interfere with the biological functions of the host gene product. The insertion of TE within exons might alter the open reading frame (ORF) and initiate missense or nonsense mutations that can consecutively destroy transcription factor binding sites [3]. By contrast, insertion into gene regulatory and coding regions can introduce new splice sites [51], perturb the canonical splice sites [52], or create new polyadenylation signals [10]. Insertions of TE into introns are also able to create alternatively spliced exons that are biologically functional [52,53]. For instance, *Alu* sequences are commonly found to create new exons in mature transcripts upon insertion [51,53]. Sorek et al. reported that more than 80% of *Alu* insertions in the exons caused either a frame-shift or premature stop codon [54]. In addition, TE insertions in 3′UTRs and introns affect mRNA stability, localization, and translation [11], causing a reduction of gene expression. Transcription factors that are commonly involved in carcinogenesis, including FOXA1 [55], SP1 [56], GATA [57], P53 [58], and retinoic acid receptors [59], have binding sites in *Alu* elements [58]. In addition, p53 binding sites have been found to interact with several LTR- and non LTR elements. It is predicted that around 400,000 p53 binding sites of the human genome are associated with the *Alu* element [58]. The deregulation of transcription factor p53 affects the expression of many other genes and is suggested to be involved in more than half of human cancers [60]. Mutations of the *TP53* gene cause an elevated activity of RNA polymerase III [61]. Levels of Alu RNA expression are increased in many cancers, including hepatocellular carcinoma and lung cancer [61]. It seems that the p53 binding to *Alu* sequences accompanied by transcriptional repression can trigger the deregulation of multiple genes [58]. It has recently been shown that p53 is able to restrain retrotransposon activity through direct interactions with the PIWI-interacting RNA pathway [62].

**Figure 2:**
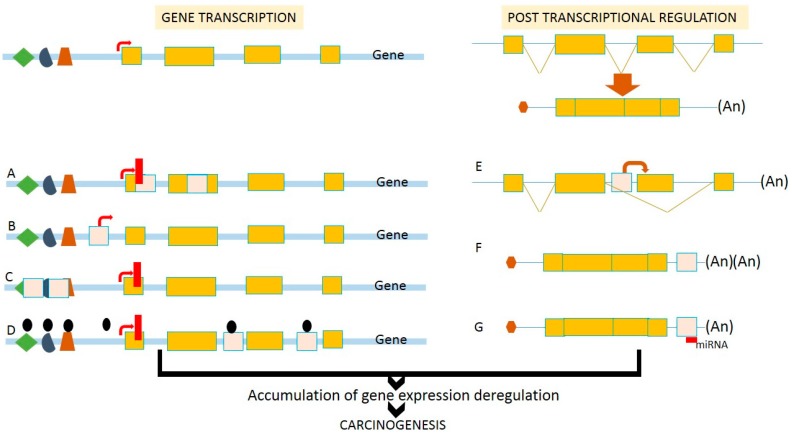
Effects of retrotransposition on transcriptional deregulation. Insertion of TEs into (**A**) coding region can disturb or eliminate gene functions; (**B**) upstream of the gene loci can introduce a novel alternative promoter leading to the variation of protein products; (**C**) promoter region can disrupt *cis*-regulatory elements, as well as transcriptional start sites; (**D**) introns can introduce epigenetic remodeling events including DNA methylation and chromatin condensation, leading to gene silencing. At the post-transcriptional step, the introduction of TEs (**E**) in the introns can cause alternative splicing that causes various protein products and functions; (**F**) at the 3′UTR can introduce poly-adenylation sites leading to unstable mRNAs; and (**G**) at the 3′-UTR can create binding sites for miRNAs and other ncRNAs. Therefore, retrotransposition affects the efficiency of gene transcription and post-transcriptional regulation, and is associated with the deregulation of gene expression during human carcinogenesis.

TE insertions within the genome are considered to be non-random events, as the insertions might affect transcriptional regulation [3,63]. It has been documented that TE integrations caused significant alterations in several *cis*-regulatory elements of gene expression [63]. A comprehensive analysis using ChIP-seq has revealed that almost 20% of transcription factor binding sites are embedded within TEs [63]. TEs are able to divide the genome into transcriptionally active or inactive regions by influencing the extent of heterochromatin formation [64]. The effects of retrotransposition on gene transcription in human cancer are summarized in Figure 2. Cancer-associated genes that are transcriptionally modulated by TE insertions are recapitulated in Table 1.

TEs also interact with non-coding RNAs, including microRNAs and lncRNAs, to modulate transcription, as discussed below. TEs use other mechanisms to influence gene expression, including the spreading of repressive chromatin signals to adjacent regions in order to silence the nearby genes [85]. In addition, TEs disrupt gene expression through chromosomal integration that can result in shorter 3′UTR and polyadenylation signal disruption [86]. The insertion of an *Alu* element into the *NF-1* gene has been shown to cause deletion and reading frame-shift, leading to the development of neurofibromatosis [87].

## 4. Genomic Instability and Chromosomal Rearrangements

The vast majority of TE insertions target particular genomic loci that are specifically recognized by endonuclease domains causing alterations of adjacent gene expression and significant genomic deletions [88]. Under certain conditions, random TE integrations can lead to insertional mutagenesis [2]. Genomic variations affected by TE mobility include the loss of heterozygocity insertion, deletion, duplication, translocation, and inversion [2,15,89]. Gene mutations caused by retrotransposon insertions are found in around 0.3% of total mutations [2]. Several mechanisms leading to TE-mediated genomic instability in cancer have been reported. First, the insertion of inverted TEs in the human genome can induce DNA damage [90]. Because TEs are naturally very mobile, there is a high chance for insertion in an inverse orientation. Several adjacent *Alu* and short sequence repeats that are inversely inserted during DNA replication tend to form a secondary hairpin structure that is susceptible to double-strand breaks (DSBs) [90,91]. However, the arrangement of inverted *Alu* elements in the human genome is relatively rare. In some parts, there is a tendency for the direct orientation of newly adjacent *Alu* insertion to prevent secondary hairpin formation [92]. Despite the unusual nature of closely inverted *Alu* insertion, the fact that unregulated *Alu* insertions frequently occur in cancer has increased the probability of de novo inverted insertions [93].

The second mechanism of TE-mediated genomic instability is the induction of unstable microsatellite seeding after TE insertions [15]. Mutations in the poly-A tail are able to initiate the seeding of large tracts of unstable microsatellite repetitive sequences [15]. If located in fragile sites of the human genomes, the unstable microsatellites can induce carcinogenesis [15]. In addition, the expansion of intronic microsatellite sequences is able to disrupt gene transcription, as shown in Friedrich’s ataxia [94]. Microsatellite instability is an important feature of several colorectal, stomach, endometrium, ovary, urinary tract, skin, and brain cancers [95]. In addition, the incorporation of TEs into introns can produce unstable long repeats in the microsatellites, causing these regions to become highly unstable and vulnerable to DNA damage [15,95].

TE-mediated genomic instability is also associated with alterations in the overall transcription in cancer. As previously mentioned, TE mobility can induce aberrant and recombinant gene transcription [58]. The interplay between the replication fork and transcription factors has significantly increased the chances of DNA damage [95]. The TE-mediated introduction of novel alternative promoters is capable of elevating the overall levels of stress-associated gene expression that may further increase DNA damage rates [89,96]. The majority of cancers represent epigenetic reprogramming with global widespread hypomethylation that can increase the transcriptional activity and mobility of these TEs, thus increasing the probability of transcriptional stress-associated DNA damage. Transcriptional stress and DNA damage are the major contributors of genomic instability in cancers [97].

The induction of genomic instability by TE insertion will induce host DNA repair signaling. The recruitment of the DNA repair machinery to the TE insertion loci is accompanied by cell cycle arrest affecting the relative efficiency of TE mobility. However, TE insertion also targets genes that are involved in the DNA repair pathway. The breast cancer susceptibility gene *BRCA2* has been affected by multiple TE insertions in cancers [12,98]. In addition, the *APC* gene is also disrupted by multiple TE insertions (*Alu* and *LINE1*) and is suggested to play a role in colorectal carcinogenesis [13,99]. Voineagu et al. have suggested that inverted repeat sequences might emerge as fragile sites inducing genomic instability [90]. LINE1 endonucleases are eminent sources of DSBs that subsequently affect genomic instability [100]. In addition, *LINE1* transposition causes DNA breakage and, even after DNA repair, this event still produces the deletion of a few base pairs [101,102]. In addition, genotoxic stress by a low dose of radiation results in *LINE1* activation and is associated with the initiation of carcinogenesis [103]. TE-associated genes that lead to deletions or variations of a few base pairs (genomic instability) in human cancer are summarized in Table 2.

TE insertions have also been associated with chromosomal rearrangement. *Alu* sequences are frequently found in several breakage points of chromosomal translocations and are closely associated with cancer [120]. TE insertion-mediated deletions and chromosomal rearrangement were reported upon pathogenic *LINE1* and *Alu* insertions [121,122]. TE-mediated insertions can cause deletions, ranging from a few to several thousand nucleotides in size [122]. Novel *LINE1* insertions often target endogenous elements, suggesting a preference for specific incorporation sites [5,121]. Chromosomal rearrangements frequently occur in rather prone regions during TE insertions. Subsequently, host DNA repair systems will try to eliminate nascent TE insertions to limit the impacts on genome instability and chromosomal rearrangement [101,123].

TE-associated chromosomal rearrangements are also mediated by segmental duplications that are relatively common in the human genome [124]. Segmental duplications have been established as significant factors in genome evolution, as well as in the development of human cancer. A comprehensive analysis of the human genome has revealed segmental duplications with an increased density of *Alu* repeats within or near the duplication intersections [124]. In humans, it is believed that an outburst of *Alu* repeats can initiate segmental duplications [124,125]. However, the exact mechanisms by which *Alu* and other TE species induce segmental duplications remain unclear. Homologous repair is able to generate duplications by arranging replicons tandemly. However, the mechanism underlying large duplication events with a distance of more than 1 Mb remains elusive. The relatively frequent *Alu* element insertions at the junction of the segmental duplication, accompanied by the involvement of evolutionarily young and identical elements, suggest that homology sequences guide these events [124,125]. TE-associated genes that are affected by chromosomal aberrations in human cancers are listed in Table 3.

## 5. Inactivation of Tumor Suppressor Genes and Activation of Oncogenes

Random integrations of TEs into specific sites of the human genome increase the chance for insertional mutagenesis followed by the activation of signaling pathways leading to carcinogenesis [2,15]. *LINE1*, *Alu*, and *SVA* are among the most common TEs that frequently induce insertional mutagenesis [2]. The activation of oncogenic drivers is also mediated by the deleterious insertion of tumor suppressor genes and the disruption of regulatory sequences. The deregulation of gene expression, splicing-induced truncated proteins, and destabilized mRNAs all contribute to oncogenic activation [3,134]. In addition, genomic instability, including chromosomal breakages and DNA recombination that can be induced by mobile TE insertions, reinforces mutation rates and carcinogenesis [13,89,127].

Non-allelic homologous recombination causing deletions or duplications in the presence of *Alu* is abundantly found in tumors with *TP53* mutations [58]. The close proximity of several *Alu* insertions tends to impose an inverted orientation, leading to the loss of p53 functions [58]. *Alu* sequences are also involved in mismatch repair (MMR) by disrupting *MLH1* and *MLH2* genes [92]. In addition, rearrangements due to *Alu* insertion and the presence of *Alu* in the MLH1 and MLH2 proteins are associated with hereditary non-polyposis colorectal cancer [92]. Recombination events due to the high density of *Alu* elements within the *BRCA1* gene are associated with the important deregulation of genomic integrity in breast cancer [12,98]. *Alu* repeats have also contributed significantly to chromosomal translocations, including *BCR/ABL* rearrangement in chronic myelogeneous leukemia [126]. The recombination of *Alu* has also caused myoblastosis (*MYB*) duplication to T-cell acute lymphoblastic leukemia [132]. Chromosomal rearrangements in mixed-lineage leukemia (MLL) have also been reported due to *Alu* recombination [135]. Especially in partial duplication events, TEs are usually inserted near the translocation breakpoints. MYB and MLL duplications have also been found in healthy controls, whereas leukemogenesis is induced by TE insertions during blood cell differentiation [136]. Additionally, *Alu* rearrangements have been reported in the tumor suppressor gene von Hipple-Lindau (VHL) [133]. Compared to oncogenes, tumor suppressor genes contain more *Alu* sequences [93].

## 6. Transposable Elements and Non-Coding RNAs

The interconnection between TEs and non-coding RNAs (ncRNAs) has been recently delineated, especially in the biogenesis of small ncRNAs, including microRNAs that are associated with TEs. MicroRNAs are small non-coding RNAs that regulate post-transcriptional gene expression and modulate several important oncogenic pathways establishing a dynamic network of cell homeostasis (reviewed in [137,138]). A large number of microRNAs originate from loci flanked from two related TEs in one genomic locus that is easily transcribed and processed into hairpin RNA structures following common microRNA biogenesis [139,140]. Both bioinformatics and genome-wide screening have identified a significant number of TE-based microRNA (up to 15% of total microRNAs) [140,141]. Several types of TEs are involved in microRNA biogenesis, especially DNA transposons, *LINE*s, and *SINE*s [142]. The first example of TE-associated microRNA is *hsa-mir*-*548* that derives from inverted-repeat transposable elements [143]. The deregulation of TE is implicated in the disruption of microRNA biogenesis. More importantly, TE-associated ncRNAs are involved in several important regulatory networks and associated with some diseases. The involvement of lncRNAs in cancers and their potential applications in clinical management of cancer have been comprehensively reviewed in [144,145].

It has also been indicated that *Alu* insertions are able to create a targeting site for mRNA decay by forming stable RNA/mRNA interactions through intra- or intermolecular base pairing with 3′UTRs and complementary binding with RNA targets [146]. Hundreds of long non-coding RNAs (lncRNAs, up tp 80%) contain endogenous insertions of TEs in human cells that serve as regulatory signals for cell growth and proliferation [147]. Numerous long circular ncRNAs are found in fibroblasts and act in a similar manner to “sponges” for miRNAs, in which intronic *Alu* sequences that flank exon and the alternative formation of inverted Alu pairs are suggested to greatly contribute to RNA circularization [148,149]. Therefore, the deregulation of TE might interrupt ncRNA functions and is associated with the initiation of carcinogenesis. *Alu* and *LINE*-embedded sequences also have regulatory roles for the transcription of lnc-RNAs and the stability of mRNA products [150]. Since lncRNAs containing repeated sequences are also important in the regulation of other epigenetic mechanisms, including genomic imprinting and chromatin remodeling, the deregulation of this network is suggested to play a role in the development of human cancer [143,150,151]. Carrieri et al. have delineated two functionally important regions in the antisense lncRNAs, i.e., 5′-end and embedded *SINEB2* and *Alu* elements that can interact with targeted mRNAs to inhibit the translation. [152]. They also identified 31 antisense lncRNAs containing similar *SINE/Alu* elements that might interact with 3′UTR mRNAs, suggesting an important role of TE-embedded regions in the post-transcriptional regulation of gene expression [152].

Reciprocally, small non-coding RNAs including microRNAs have also been reported to regulate genomic stability through direct or indirect transcriptional and post-transcriptional TE repression [150]. As cytoplasmic ncRNAs, microRNAs indirectly control DNA repair and genome stability [153]. siRNAs mediate the *trans* enrolment of Ago complexes to subsequently regulate genome instability through transcriptional repression and repetitive DNA element recombination [153]. In addition, the microRNA biogenesis machinery has been inferred as an important regulator of heterochomatin formation and transcriptional silencing. The AGO1 protein is reported to be involved in transcriptional gene silencing through histone H3K9 methylation [154]. An initial report suggested that promoter DNA methylation causing transcriptional silencing can be stimulated by a complementary siRNA to direct DNA methylation into specific loci, including repetitive sequences [155]. Another mechanism of ncRNA deregulation is the insertion into microRNA’s regulatory regions. *Alu* sequences occupy a large portion of microRNA genes, as well as 3′UTR mRNA targets [156]. In general, tumors are able to avoid miRNA-mediated regulation, causing a further enhancement of genomic instability and mutability because of TE reactivation.

LncRNAs have been associated with myriads of important signaling pathways, most of which require an interaction with proteins, as well as transcription factors. TE-associated lncRNAs are reported to bind to some important transcription factors for cell proliferation, including p53, sp1, and NF-Y [157]. In response to cellular stress, Alu RNAs are able to bind to RNA polymerase II to regulate the expression of some responsive genes, as well as to promote evolution through exonization and alternative splicing [158]. Analysis using mass spectrometry has revealed that *Alu*-derived piRNAs are able to bind some nuclear proteins, suggesting their potential roles in DNA repair, chromatin reprogramming, and cell proliferation [159]. Therefore, TE mobility within lncRNA transcripts reflects evolutionary and versatile functions in regulating cellular homeostasis.

The PIWI-piRNA axis has also been implicated in the silencing of transposable elements and contributes to the development of human cancers [160]. Transcribed mostly from a cluster, piRNAs are small non-coding RNAs that interact with PIWI proteins that are abundantly found in germ cells [161]. PIWI proteins interact with DNA methylation and chomatin modifications to silence TEs while maintaining the ability of germ stem cells for self-renewing [162]. In addition, the piRNA pathway interacts with the p53 protein to restrict mobile element insertions and regulate chromatin repressive marks in the 5′ of *LINE1* sequences [62]. One of the most important roles of the piRNA-PIWI protein complex is to protect germ cells from transposon insertions [160,163]. In cells that are previously exposed to TEs, piRNAs containing a complementary TE sequence will induce degradation through Piwi proteins [164]. In the first encounter to TEs, piRNA transcripts that are complementary to the TE RNA can induce degradation through the piRNA-Piwi complex [165]. The piRNA-Piwi axis has also been associated with the maintenance of genomic imprinting through DNA methylation in the Rasgfr locus, in which the differentially methylated region contains the *LINE1* sequence [166]. Correspondingly, the deregulation of PIWI proteins and transposable elements has been reported in gliomas, sarcomas, and adenocarcinomas [160].

## 7. Conclusions

**Figure 3:**
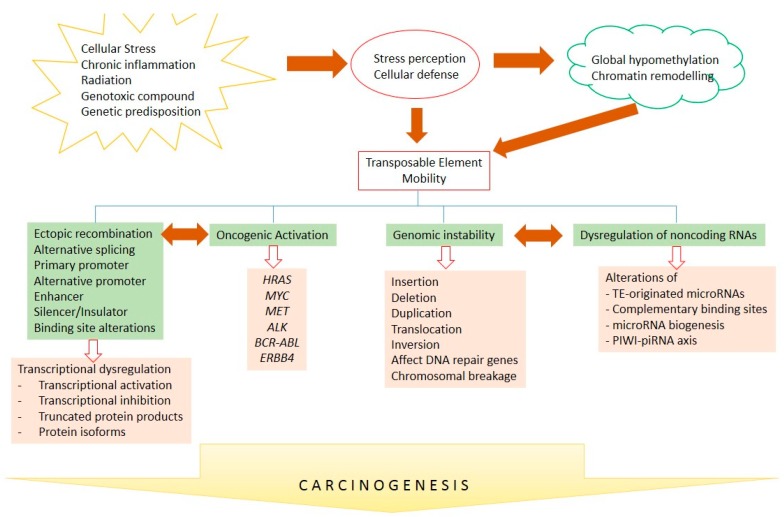
TE-mediated carcinogenesis. Intra- and extracellular-mediated stresses lead to TE mobility through alterations of DNA methylation and chromatin remodeling. TE mobility might further induce and interconnect transcriptional deregulation, the activation of oncogenes, genomic instability, and ncRNA deregulation, to further contribute to human carcinogenesis. Arrows show causality and bidirectional arrows represent inter-correlation.

The above compiled results and observations suggest an important role of TEs in transcriptional control, genomic instability, chromosomal rearrangements, non-coding RNA regulation, and oncogenic activation. Although a direct association between TE insertion and cancer initiation still has to be clarified, the deregulation of TEs is closely linked to cancers of various etiologies. Bioinformatic screens have revealed mutagenenic TE insertions in different types of cancers. TE insertions also function as a reservoir of endogenous gene regulatory factors that have been co-opted by the human genome to control gene expression, as well as cellular phenotypes. After co-option or exaptation, insertions of TEs can provide novel transcription factor binding sites in the promoters, enhancers, or insulators, leading to cancer specific activation. TEs flanking ncRNA genes are also involved in ncRNA biogenesis. In addition, repetitive sequences within TEs are important loci for the complementary binding of ncRNAs acting as a reservoir for ncRNA inactivation. Therefore, the deregulation of TEs affects genomic stability, transcription, and non-coding RNA regulation, leading to cancer development and progression (please see Figure 3). However, the mechanisms for TE-mediated oncogenic activation need to be further investigated to reveal new insights into carcinogenesis and identify novel targets for therapeutic interventions. Clarification of the specific epigenetic regulation of TEs in such studies may raise the possibility of applying epigenetic agents in TE-driven cancers such as demethylase and bromodomain inhibitors; that are now in preclinical and clinical trials for solid tumors and hematological malignancies.

## Figures and Tables

**Table 1:** TE-associated transcriptional deregulation in human cancers.

Locus or Genes	TE(s)	Mechanisms	Associated Cancers	References
*ADARB1*	*Alu*	Alternative splicing	Lung, brain cancer	[65,66,67]
*AKR1A1*	*Alu*	Alternative splicing	Head and neck cancer	[65,68]
*ALS2CR8*	*LINE1*	silencing	Colorectal cancer	[2]
*ANKS1B_*	*Alu*	Deletion, silencing	Colorectal cancer	[2]
*ANO9*	*LINE1*	Alternative splicing	Colorectal cancer	[2]
*ADH1C*	*LTR*	Primary promoter	Cancers	[65,69]
*ALK*	*LINE1, LTR*	Alternative promoter	Melanoma, cancers	[17]
*APOA*	*LINE1*	Enhancer	Cancers	[65,70]
*APOC*	*LTR*	Alternative promoter	Gastric cancer	[65]
*ARNT*	*Alu*	Alternative splicing	Lung cancer, metastasis of different cancers	[65]
*ARHGEF12*	*LINE1*	Alternative splicing	Ovarian cancer	[2]
*ASMT*	*LINE1*	Alternative splicing	Glioma	[65]
*B3GALNT2*	*Alu*	Alternative splicing	Breast cancer	[65]
*B3GALT5*	*Alu*	Alternative promoter	Breast cancer	[65]
*BAAT*	*LTR*	Primary promoter	Lung cancer	[65]
*BAHCC1*	*Alu*	Alternative splicing	Colorectal cancer	[65]
*BBS7*	*LINE1*	Primary promoter	Prostate cancer	[2]
*BLVRA*	*Alu*	Alternative splicing	Breast cancer	[65]
*C19MC miRNA*	*Alu*	POL III promoter	Hepatocellular, human cancers	[35,65,71]
*CA1*	*LTR*	Primary promoter	Colorectal cancer	[8,72]
*CASPR4*	*LTR*	Alternative promoter	Breast cancer, nasopharyngeal cancer	[65]
*CD8A*	*LTR*	Enhancer	Colorectal, pancreatic cancer	[65]
*CDH12, CDH20*	*LINE1*	Alternative splicing	Colorectal cancer	[2]
*CHRM3*	*LINE1*	Alternative promoter	Endometrial cancer	[65]
*CHRNA1*	*Alu*	Alternative splicing	Testicular cancer	[65]
*CLDN14*	*LTR*	Alternative promoter	Hepatocellular carcinoma	[65]
*COL11A1, COL9A1*	*LINE1*	Alternative splicing	Colorectal, prostate cancer	[2]
*COX7B2*	*LINE1*	Alternative splicing	Prostate cancer	[2]
*CTNNA2*	*LINE1*	Alternative splicing	Colorectal cancer	[2]
*CWF19L1*	*Alu*	Alternative splicing	Breast cancer	[65]
*CYB561D1*	*Alu*	Alternative splicing	Lung cancer	[65]
*CYP19A1*	*LTR*	Alternative promoter	Breast cancer	[65]
*CUL3*	*LTR*	Alternative splicing	Lung cancer	[73]
*DAPK1*	*ERV1*	Alternative splicing	Multiple myeloma	[2]
*DBC1*	*LINE1*	Deletion, Alternative promoter	Colorectal cancer	[2]
*DHRS2*	*LTR*	Alternative promoter	Breast cancer	[65]
*DNMT1*	*Alu*	Alternative splicing	Different cancers	[65,74]
*DSCR4, DSCR8*	*LTR*	Primary promoter	Colorectal cancer	[65]
*EPHA6*	*LINE1*	Alternative transcript	Colorectal cancer	[2]
*ERBB4*	*LTR*	Alternative promoter	Lymphoma, colorectal cancer	[2,19]
*EBR*	*LTR*	Alternative promoter	Bladder cancer	[65]
*FABP7*	*LTR*	Alternative promoter	Lymphoma	[18]
*FLT4/VEGFR3*	*LTR*	Alternative splicing	Different cancers	[65]
*FMO1*	*LINE1*	Silencer	Lung cancer	[65]
*FOXP2*	*LINE1*	Alternative promoter	Ovarian cancer	[2]
*FUT5*	*LINE1, Alu*	Alternative splicing	Colorectal cancer	[65]
*GABRG3*	*LINE1*	Alternative splicing	Colorectal cancer	[2]
*GBP5*	*LTR*	Primary promoter	Breast cancer	[65]
*HHLA2, HHLA3*	*LTR*	Polyadenylation signal	Bladder cancer	[65]
*HINFP/ZNF743*	*Alu*	Alternative splicing	Lung cancer	[65]
*HYAL-4*	*LINE1, Alu*	Primary promoter	Cancers	[8,75]
*IFNγ*	*Alu*	Binding sites	Cancers	[65]
*KCNH6*	*Alu, LTR*	Alternative splicing	Endometrial cancer	[76,77]
*KDR*	*LINE1*	Alternative promoter	Colorectal cancer	[2]
*MCTP2*	*LINE1*	Deletion, Alternative promoter	Colorectal cancer	[2]
*MET*	*LINE1*	Alternative splicing	Bladder cancer	[16]
*MKKS*	*LTR*	Alternative promoter	Colorectal cancer	[65]
*MSLN*	*LTR*	Primary promoter	Pancreatic cancer	[65]
*NAIP*	*Alu*	Alternative promoter	Cancer	[78]
*NFKBID*	*Alu*	Alternative splicing	Colorectal cancer	[65]
*NOS3, NOSIP*	*LR*	Alternative promoter	Different cancers	[65]
*NPAS3*	*LINE1*	Alternative promoter	Colorectal cancer	[2]
*NRXN3*	*LINE1*	Alternative splicing	Colorectal cancer	[2]
*RB1*	*LTR*	Alternative promoter	Hepatocellular carcinoma, retinoblastoma	[14,79]
*ROBO2*	*LINE1*	Alternative splicing	Colorectal cancer	[2]
*SLCC44A5, SLC35F1*	*LINE1*	Alternative promoter, slencing	Colorectal cancer	[2]
*SRY*	*LTR*	Alternative transcript	Wilm’s tumor	[80,81]
*STXBP5L*	*LINE1*	Alternative promoter	Colorectal cancer	[2]
*TMED7*	*Alu*	Alternative promoter	Colorectal cancer	[2]
*TMEM16J, TMEM56*	*Alu*	Alternative splicing, silencing	Colorectal cancer	[2]
*TMPRSS3*	*Alu, LTR*	Alternative transcript	Breast, ovarian cancer	[8,82,83]
*TP53*	*Alu*	Binding sites	Cancers, Pancreatic cancer	[58]
*P63*	*LTR*	Primary promoter	Breast cancer	[65]
*PDZK1*	*Alu*	Alternative splicing	Lung cancer	[65]
*PODXL*	*Alu*	Alternative splicing	Pancreatic cancer	[65]
*PTN*	*LTR*	Alternative promoter	Different cancers	[65]
*ZNF451*	*LTR*	Alternative splicing	Hematological cancer	[9,84]
*ZNF177, ZNF257, ZNF418*	*LINE1, Alu*	Alternative splicing	Different cancers	[65]

**Table 2:** TE insertion-associated loss or gain of a few base pairs and mutagenesis in human cancers.

Locus or Genes	TE(s)	Associated Cancers	References
*APC*	*Alu*	Colon cancer	[13,99]
*BRCA1*	*Alu*	Breast, ovarian cancer	[12,98,104]
*BRCA2*	*Alu*	Breast, ovarian cancer	[12,98,104]
*CASPR4*	*LTR*	Brain cancers	[8,105]
*CHEK2*	*Alu*	Breast, ovarian, prostate cancer	[104,106]
*CLDN14*	*LTR*	Melanoma	[8,107]
*CYP19*	*LTR*	Breast cancer	[8,108]
*ENTPD1*	*LTR*	Melanoma	[8,109]
*FUT5*	*LINE1, Alu*	Gastric cancer	[8,110]
*HSD17B1*	*LTR*	Breast, endometrial cancers	[111]
*HRAS*	*LTR*	T-cell leukemia	[112]
*MLH1*	*Alu*	Colorectal cancer	[2,113]
*MLVI2*	*Alu*	Leukemia	[114]
*MKKS*	*LINE2, LTR*	Embryonic cancers	[8,115]
*MLH2*	*Alu*	Colorectal cancer	[113]
*MYC*	*LINE1*	Breast cancer	[116]
*NF1*	*Alu*	Neurofibromatosis type I	[87]
*MSLN*	*LTR*	Human cancers	[8,117]
*RB1*	*Alu, LTR*	Retinoblastoma, hepatocellular cancer	[14,79]
*TCF3-PBX1*	*LTR*	ALL	[118]
*TRPC6*	*LTR*	Breast, prostate, gastric cancers, glioma	[76,119]

**Table 3:** TE-mediated chromosomal structure defects in human cancers.

Locus of Genes	TE(s)	Chromosomal Defects	Associated Cancer	References
*BCR-ABL*	*Alu*	Chromosomal translocation	CML	[126]
*BRCA1*	*Alu*	Chromosomal deletion, duplication, insertion	Breast, ovarian cancer	[127]
*BRCA2*	*Alu*	Chromosomal deletion, duplication, insertion	Breast, ovarian cancer	[12,128]
*CAD*	*Alu*	Chromosomal deletion	Hepatocellular carcinoma	[122]
*CDH1*	*Alu*	Chromosomal deletion	Diffuse gastric cancer	[129]
*EWSR1-ETV*	*Alu, LINE1*	Chromosomal translocation	Ewing sarcoma	[28]
*MAD1L1*	*LTR*	Chromosomal instability	Breast cancer	[8,130]
*MLL1*	*Alu*	Chromosomal duplication	AML	[131]
*MYB*	*Alu*	Chromosomal duplication	T-ALL	[132]
*VHL*	*Alu*	Chromosomal deletion	von Hippel Lindau disease	[133]
*WT1*	*LINE1*	Chromosomal translocation	Sarcoma	[28]
